# The Mole: Its Relation to Malignant Diseases of the Skin

**Published:** 1912-10

**Authors:** Jos. Spangenthal

**Affiliations:** Buffalo, N. Y.


### The Mole: Its Relation to Malignant Diseases of the Skin. A Plea for Its Removal.

By JOS. SPANGENTHAL, M.D.
Buffalo, N. Y.

THE author imbued with the importance of the subject presents it for the consideration of the medical profession in general. To the specialist in dermatology it is almost necessary to apologize for offering a subject the importance of which has been so thoroughly recognized.

It appears trivial to urge the removal of an innocent growth like the mole, and justly so if removed for cosmetic purposes only; but, when consideration is given how frequently the dermatologist encounters malignant diseases of the skin, such as Epithelioma and Sarcoma in which a history is obtainable of an antecedent mole, then does this innocent blemish merit our most earnest thought.

After middle age certain changes occur in the skin. The skin at this time loses its elasticity, becomes relaxed and wrinkled. The hair which is a dermal appendage participates also in these changes, in becoming lusterless and gray. These are physiological changes due to age.

Concomitant changes are likewise observed in the mole, which are not always physiological. Oftentimes, there is seen a slightly scaly degeneration, which is distinctly pathological. Such a condition might exist for years without causing any anxiety, but too frequently it is otherwise.

There may be an increase in pigmentation, an hypertrophy of the papillæ, a border waxy in appearance with capillary dilitation, and here we have a picture of epithelioma. This condition might remain stationary for long periods of time, but is usually progressive. Infiltration with subsequent ulceration occurs, until there is an involvement of all the structures of the skin and underlying tissues.

It is surprising how frequently patients present themselves to the dermatologist with epitheliomata in all stages of development, who were not aware of the seriousness of their condition. They give a history of a wart or mole which had been present for years; and only after serious degenerative changes have occured, do they consult the dermatologist.

Too often does the family physician who perhaps has seen it in its incipiency only, pacify the patient with assurance of its insignificance. The patient's anxiety appeased, considerable progress would occur, and valuable time would be lost.

The following two cases will suffice as illustrations. Case 1. A middle aged man suffering from a large melanotic carcinoma, in which the large toe and part of the foot were involved. The history was that of a pigmentary mole of many years duration. This began to undergo changes of a malignant nature, which caused him some alarm. Still he did not think it serious enough to consult a dermatologist. The patient neglected for some time, and later came to the city for consultation. A diagnosis of melanotic carcinoma was made, and a complete amputation of the foot was necessary. A year previous, a simple operation for the removal of the mole would have been sufficient.

Case 2. A woman aged 65, with a malignant growth the size of a cherry, situated on the end of her nose. This same patient had an epithelioma over the tibia, another the size of a penny on her abdomen, a nævus pigmentosus undergoing epithelial degeneration on her chest, and another nævus over the right temporal region. These fortunately were amenable to successful treatment.

In an address delivered by Dr. W. S. Bainbridge, Surgeon to the N. Y. Skin and Cancer Hospital—"The Campaign Against Cancer, Educational, Experimental and Clinical," he says: "Actual experience gained from the study of hundreds of cases similar to those presented above, (See *American Journal of Dermatology*, Vol. XV., No. 7) has convinced us that a large proportion of severe and perhaps fatal malignant neoplasms may be traced to apparently insignificant and harmless warts, moles, nævi, scars, etc., which are subjected to irritation of one kind or another, and which are easily and completely removed by surgical means."

Hartzell in an excellent paper entitled "Some Precancerous Affections of the Skin," writes, "New growths of the skin congenital or acquired after a longer or shorter duration as benign affections, may undergo cancerous change; warts and pigmented nævi showing the greatest tendency to such a metamorphosis." Again he states that of 223 cases of carcinoma of the extremities collected by Rudolph Volkmann, 23 or a trifle more than 10% had their origin in congenital or acquired warts or moles. He also writes of the pigmented epithelioma originating from the pigmented nævus as being the most malignant of the new growths; of its rapid advancement and wide spread metastases and its almost certain recurrence.

In making a plea for the removal of the mole, two reasons are worthy of consideration, the cosmetic and the prophylactic. The operation for cosmetic purposes, applies to all young individuals; for among them, malignancy is quite rare. In those past middle age, it becomes more urgent from a prophylactic view-point, and in these cases the operation should be advised. Especially is this applicable in the pigmentary variety.

The simplicity of the operation, and the freedom from pain and danger, offer excellent reasons for their early removal.

595 Lafayette Ave.

